# uPARAP/Endo180: a multifaceted protein of mesenchymal cells

**DOI:** 10.1007/s00018-022-04249-7

**Published:** 2022-04-22

**Authors:** Fabrice Gucciardo, Sébastien Pirson, Louis Baudin, Alizée Lebeau, Agnès Noël

**Affiliations:** Laboratory of Tumor and Development Biology, GIGA-Cancer, Liege University, B23, Avenue Hippocrate 13, Sart-Tilman, B-4000 Liege, Belgium

**Keywords:** MRC2, Cancer, Tissue remodeling, uPARAP/Endo180, Collagen, Endocytic receptor

## Abstract

The urokinase plasminogen activator receptor-associated protein (uPARAP/Endo180) is already known to be a key collagen receptor involved in collagen internalization and degradation in mesenchymal cells and some macrophages. It is one of the four members of the mannose receptor family along with a macrophage mannose receptor (MMR), a phospholipase lipase receptor (PLA2R), and a dendritic receptor (DEC-205). As a clathrin-dependent endocytic receptor for collagen or large collagen fragments as well as through its association with urokinase (uPA) and its receptor (uPAR), uPARAP/Endo180 takes part in extracellular matrix (ECM) remodeling, cell chemotaxis and migration under physiological (tissue homeostasis and repair) and pathological (fibrosis, cancer) conditions. Recent advances that have shown an expanded contribution of this multifunctional protein across a broader range of biological processes, including vascular biology and innate immunity, are summarized in this paper. It has previously been demonstrated that uPARAP/Endo180 assists in lymphangiogenesis through its capacity to regulate the heterodimerization of vascular endothelial growth factor receptors (VEGFR-2 and VEGFR-3). Moreover, recent findings have demonstrated that it is also involved in the clearance of collectins and the regulation of the immune system, something which is currently being studied as a biomarker and a therapeutic target in a number of cancers.

## Introduction

In eukaryotic cells, the extracellular and subcellular localization of a protein is tightly controlled and closely linked to its functions. In fact, distinct extracellular and intracellular compartments provide specific chemical environments (for instance pH and redox conditions) that are fundamental to potential interactions with partners or substrates. Accordingly, the management of protein subcellular localization plays a vital role in protein regulation. In this context, endocytosis is a key biological process, through which cells internalize macromolecules and cell surface proteins. The cellular uptake through one of the multiple endocytic pathways is followed by routing through the endosomal network to a final destination. Cargoes can be recycled back to the plasma membrane, sent to the trans-Golgi network (TGN) via retrograde traffic, or sorted to the lysosome where they are degraded [1, 2]. This endocytic process defines the quality of cell response to extracellular stimuli by regulating cell surface receptor clearance. It additionally contributes to tissue remodeling and cell–matrix interaction through a controlled uptake of extracellular matrix (ECM) components and integrins, their most abundant cell surface receptors [3]. As a major ECM constituent, different types of collagen are critical for tissue architecture, and form a scaffold for cell adhesion and migration. Together, these play a crucial role in regulating cell functions during embryonic development and physiopathology. Among endocytic receptors, the urokinase plasminogen activator receptor-associated protein (uPARAP) or Endo180, the product of the *MRC2* gene, is a master regulator of collagen turnover. However, beyond its collagen binding and internalization functions, it has been demonstrated that uPARAP is assigned other functions related to cell migration involved in tissue repair (wound healing) [4, 5], cancer progression [6, 7], and more recently in pathological lymphangiogenesis [8]. In this review, the main features of uPARAP/Endo180 will be presented along with its newly identified functions which are subsequently discussed.

## uPARAP/Endo180 discovery: two names, one protein

A study on membrane proteins, conducted by Isacke et al. in 1990, [9] discovered a 180 kDa protein in cultured human fibroblasts thereafter referred to as p180. The largest part of this new protein (70% to 80% of the total) was found to be present in the membrane of intracellular vesicles, which suggested that this transmembrane protein undergoes internalization and de facto enters the endocytosis circuit. It was observed that its membrane exposure could be restored, indicating that it was possible for p180 to be internalized and then, at a later stage, recycled back to the surface. During this process, the protein remained unaltered, making it a constitutively internalized and recycled protein. Similarities were then established with other well-known membrane proteins, such as the transferrin receptor (TfR), previously established as a prototype for clathrin-dependent endocytosis [10, 11]. The protein was initially synthesized as a 150 kDa single polypeptide backbone, then matured into its final 180 kDa protein form by glycosylation (N-linked carbohydrates) and the addition of neuraminidase sensitive terminal sialic acid residues. It is worth noting that no less than 75 kDa was exposed to the extracellular medium [9, 12]. This p180 protein was also found to be a substrate for the Protein Kinase C (PKC), targeting putative serine residues, as found on the TfR [13, 14]. Consequently, K. Wu et al. [15] later identified the p180 murine ortholog, as a carbohydrate calcium-dependent binding protein. Based on sequence similarities, it was, therefore, classified as a member of the mannose receptor (MR) family.

In 2000, further investigations of human p180 protein biological functions were conducted, simultaneously, by two independent research groups. Both studies shed light on the high sequence homology between human p180 cDNA and its murine ortholog, confirming its classification as a definitive and ultimate mannose receptor family member [12, 16]. The initial study investigated the cell distribution and endocytic properties of human p180, and renamed it Endo180. Gene mapping localized the *Endo180* encoding gene on the human 17q chromosome which was seen to be expressed in macrophages, stromal and endothelial cells [12]. A second study reported that only a fraction of the protein was engaged in a trimolecular non-covalent complex formation with the urokinase plasminogen activator (uPA) and its receptor (uPAR), both related to the plasminogen activation cascade. For this reason, it was renamed the urokinase-plasminogen activator receptor-associated protein (uPARAP). uPARAP/Endo180 is a receptor in a majority of type V collagen and to a lesser extent for other collagen including types I and IV. After binding with uPARAP, collagen is endocytosed into clathrin-coated vesicles and routed to early endosomes where it is dissociated from its receptor, following which the uPARAP is recycled back to the cell surface, while the collagen fragments are directed towards the lysosomal compartment for degradation [16]. In addition to its common appellations, the protein may also be found under the name of CLEC13E, KIAA0709, CD280 or TEM9. Despite sharing some similarities with other MR family members, uPARAP/Endo180 displays unique properties related to cell membrane-associated protein trafficking and cell migration that are discussed as follows.

## uPARAP/Endo180 domains and structure

**Fig. 1:**
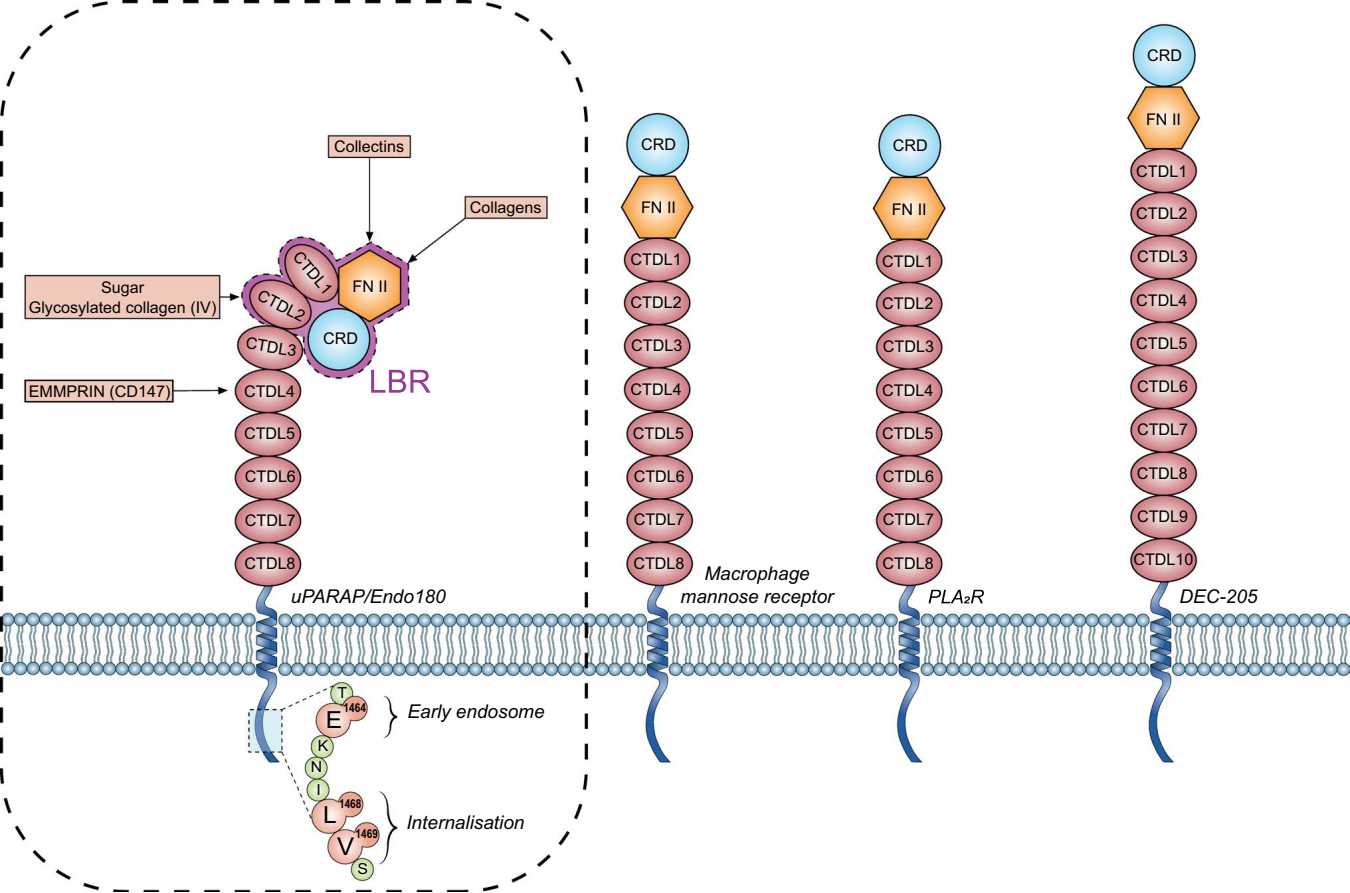
uPARAP/Endo180 belongs to the mannose receptor family. Schematic representation of the fourth members of the mannose receptor family. They share common domains from N-terminal to C-terminal including a Cysteine-rich domain (CRD), a Fibronectin type-II domain (FNII), several C-type lectin-like domains (CTLDs), a transmembrane domain and a cytosolic tail. uPARAP/Endo180 can interact with collagens, collectins and sugars or glycosylated proteins through the ligand-binding region (LBR composed of CRD, FNII, CTLD1 and CTLD2) and with EMMPRIN (CD147) through CTLD4. uPARAP/Endo180 contains two acidic residues (L1468 and V1469) responsible for its internalization and another one (E1464) controlling its trafficking

Based on its protein sequence, uPARAP/Endo180 has been assigned to the clearly defined mannose receptor family, making it the final member of the group (Fig. 1). This endocytic receptor family is made up of four type-1 transmembrane proteins: (i) the eponymous family founding member, macrophage mannose receptor (MMR, encoded by the gene MRC1) [14, 15], (ii) the phospholipase A2 receptor (PLA_2_R, encoded by the gene PLA2R1) [16, 17], (iii) dendritic receptor (DEC-205/gp200-MR6, encoded by the gene LY75) [17, 18] and (iv) uPARAP/Endo180 (encoded by the gene MRC2) [19]. MMR and uPARAP/Endo180 are the most similar members of the group and share an ability to bind and internalize collagen [20]. From the N-terminus to C-terminus, their extracellular regions are made of a peptide signal, a cysteine-rich domain (Cys-R), a fibronectin type-II (FNII) domain and several C-type lectin-like domains (CTLD) that are repeated 8 or 10 times in tandem. Within its transmembrane region, the cytoplasmic tail of uPARAP/Endo180 is responsible for receptor internalization involving a critical dihydrophobic Leu^1468^/Val^1469^ motif mediating the recruitment of the receptor into clathrin-coated pits [21, 22]. Although conserved tyrosine in a consensus sequence (FxNxxY) is crucial for the endocytosis of MMR, PLA2R and DEC-205, this residue has no effect on uPARAP/Endo180 internalization [21]. However, an acidic residue (E^1464^) is involved in uPARAP/Endo180, targeting endosomes. Given the lack of the three acidic motifs at the site (EDE present in DEC-205, a motif known to target protein to a late endosomal/lysosomal compartment), uPARAP/Endo180 is targeted to early endosomes, as is the case for the MMR [21, 23]. uPARAP/Endo180 is rapidly recycled (5 to 20 min) [7, 24] and is a relatively stable protein with an average half-life of 24 h [9]. Beyond the residues involved in protein internalization, the tail contains several phosphorylation sites whose function remains unclear. Constitutively internalized, uPARAP/Endo180 is able to internalize its ligand, another characteristic it shares with the MMR, or it simply acts as a signaling pathway modulator as described below [22].

The four N-terminal domains (Cys-R, FNII, CTLD1 and CTLD2) form an area known as the ligand-binding region (LBR), which makes the protein able to bind collagens and carbohydrates (Fig. 1). The Cys-R domain of uPARAP/Endo180 has a unique conformation that differentiates it from the typical β-trefoil fold characteristic of other MR family members [25]. Some studies using single particle electron microscopy revealed that this Cys-R domain intimately contacts the CTLD2 adopting either a L-shape, which moves the two domains closer together, or a more open conformation depending on the pH [26, 27]. One study, however, using small-angle X-ray scattering, challenged the existence of such a structural modification [28], while a second study confirmed the findings, albeit to a lesser extent and with less impact of pH on the open conformation. In this study, both CTLD1 and CTLD2 domains adopt a characteristic tridimensional CTLD structure at the cell surface (at neutral pH), with two Ca2 + in the CTLD2 and were, therefore, able to bind carbohydrate molecules [29] such as mannose, fucose and N-acetylglucosamine [30], as well as highly glycosylated collagens including type IV collagen [31]. Once it had been internalized in the cell by endosomes, uPARAP/Endo180 found itself in an acid environment with a low Ca2 + concentration and became protonated. This modification induced a CTLD2 conformational change and the release of the bound molecules. It is believed that other mechanisms of collagen release exist but this still needs to be elucidated [21, 29, 30].

The most conserved extracellular domain in the MR family is FNII, also present in some other proteins such as matrix metalloproteinases (MMPs: MMP-2 and MMP-9) [24]. It is the principal site of collagen binding in MMR and uPARAP/Endo180 [20]. Although the LBR structure is important for FNII function, it is not necessarily impacted by the conformational modification described above [28, 31]. Of the 28 existing collagen types, uPARAP/Endo180 is the one that is able to bind fibrillar collagens including types I, II and V through its FNII domain. Binding to the glycosylated basement membrane-associated type IV collagen, on the other hand, is enhanced by the CTLD2 domain [24, 31–34]. Interestingly, this FNII domain was also seen to be involved in the interaction between uPARAP/Endo180 and the uPAR/pro-uPA complex (see below). It is worth mentioning that interaction with type V collagen, prevented this complex formation [16]. Besides these important roles of FNII and CTLD2 domains of the LBR, CTLD4 contributed to the interaction of uPARAP/Endo180 with CD147/Basigin/EMMPRIN, a member of the immunoglobulin superfamily participating in tumor development [35] (Fig. 1). These data highlight the capacity of uPARAP to interact with multiple cell-associated proteins.

## uPARAP/Endo180: an endocytic receptor of collagen and collectins

**Fig. 2:**
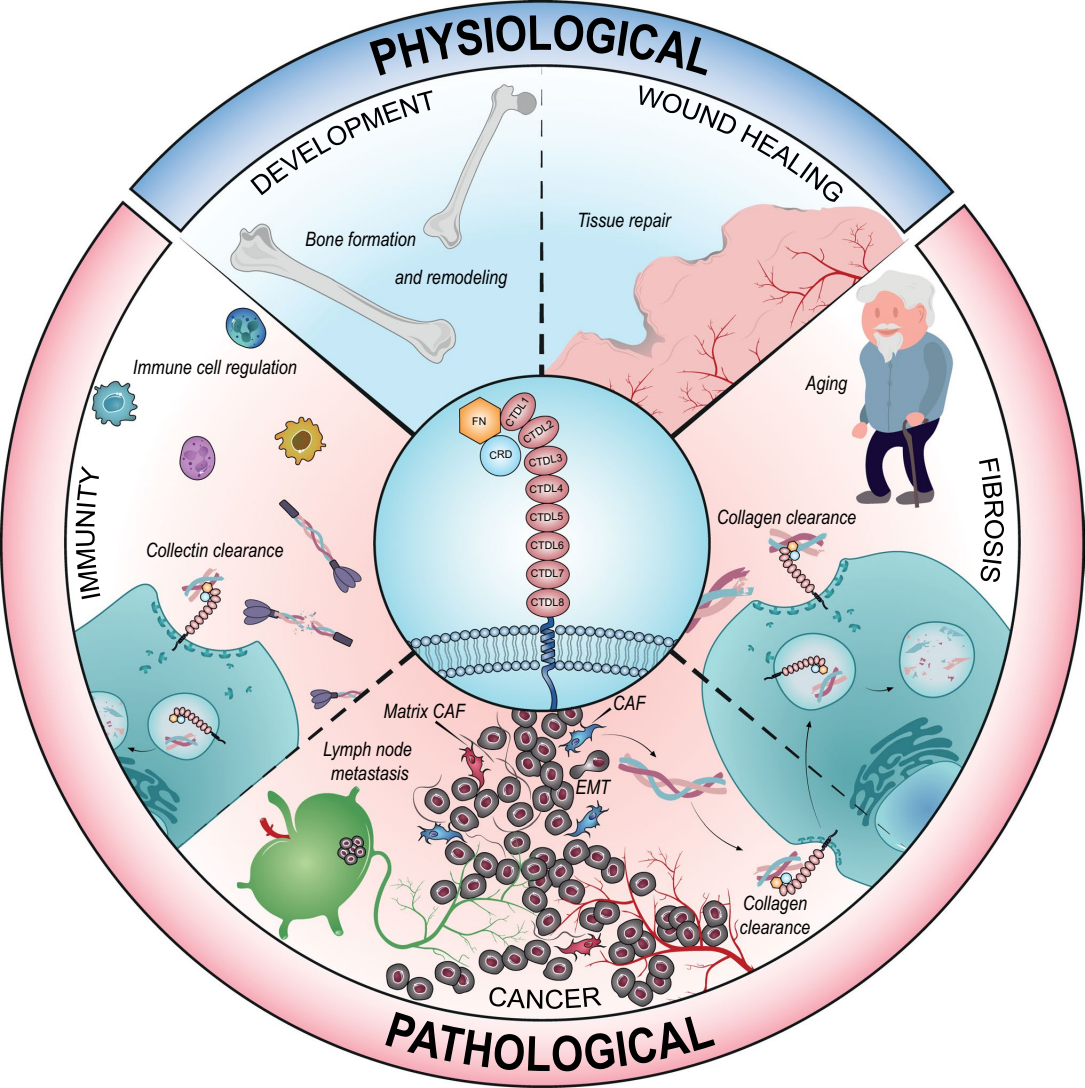
The involvement of uPARAP/Endo180 in physiologic and pathological conditions. uPARAP is involved in various physiological processes such as bone development and wound healing. At the pathological level, uPARAP involvement is implied in fibrosis through collagen clearance, in cancer and in immunity

**Fig. 3:**
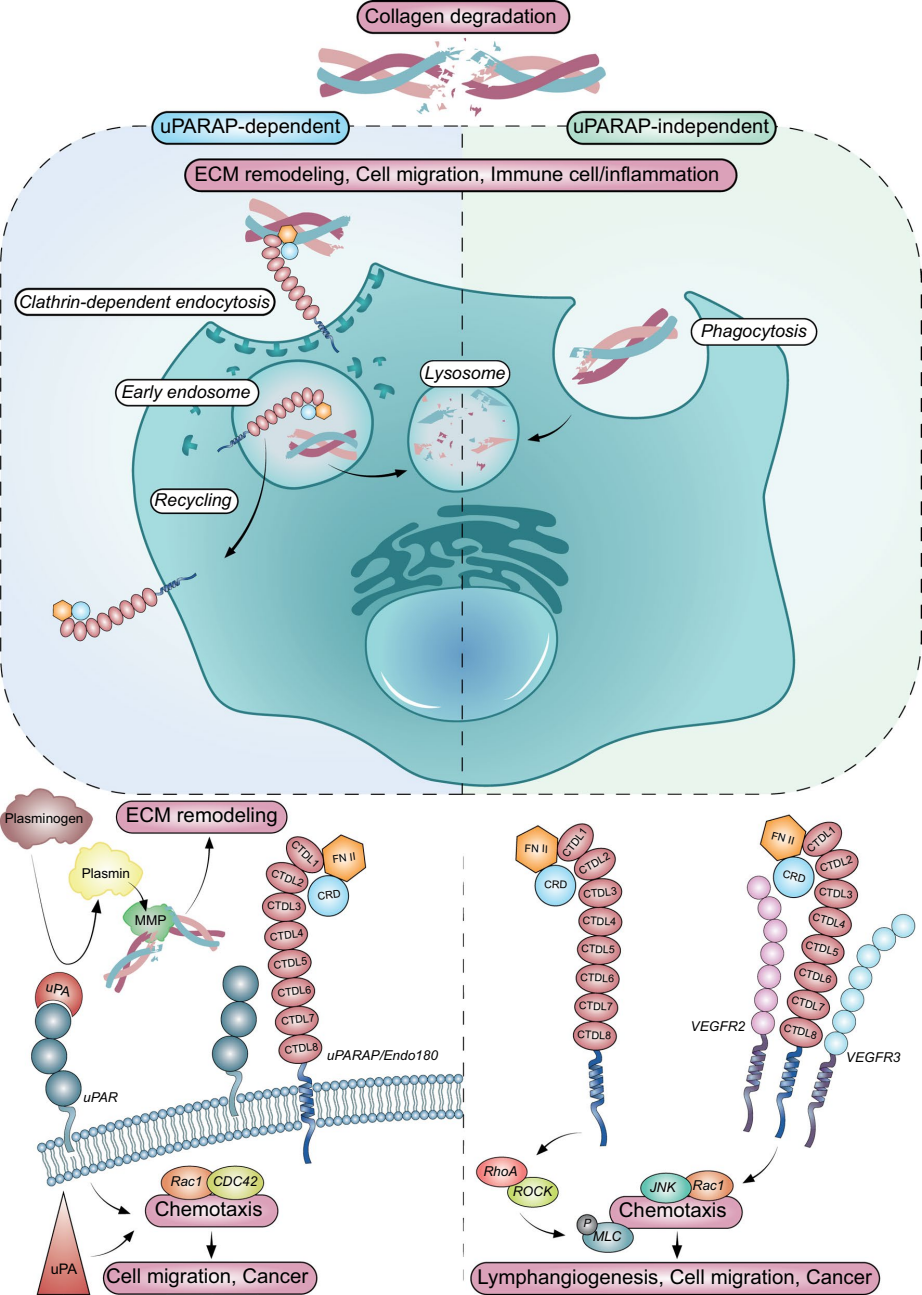
The cellular functions of uPARAP/Endo180. uPARAP is involved in extracellular matrix (ECM) remodeling through direct binding of collagen to uPARAP. Collagen bound to CTLD2 is internalized by a clathrin-dependent endocytic process. Once in the early endosome, uPARAP is recycled to the membrane while the collagen is degraded in the lysosome. The uPARAP-uPA-uPAR complex enables the conversion of plasminogen to plasmin, which results in the degradation of ECM components. This trimolecular complex is also involved in cell motility via the activation of Rac1 and Cdc42, two small Ras GTPases. uPARAP prevents the heterodimerization of VEGFR-2/VEGFR-3 ensuring the maintenance and integrity of the lymphatic vasculature

The ECM turnover involved in homeostatic maintenance and tissue remodeling takes place under physiological (embryonic development, wound healing, tissue repair) and pathological conditions (cancer, inflammation) (Fig. 2), and uPARAP/Endo180 plays its part in this [36], also contributing to the intracellular degradation of collagen with its ability to internalize its fragments. Consistently internalized at the cell steady state, uPARAP/Endo180 is a key protein in the non-phagocytic collagen uptake pathway that routes it to the lysosomal compartment (Fig. 3). As mentioned above, the low endosomal pH triggers receptor-ligand disruption, leading to the receptor recycling back to the membrane and ligand degradation [37, 38]. The clathrin-dependent endocytosis, through which uPARAP/Endo180 is transported into the cell, engulfs proteins resulting in an endocytic vesicle (endosome) with a diameter of less than 200 nm [39]. Collagen fibrils are oversized and, therefore, cannot be carried into the cytosol in a clathrin-dependent way [40]. Due to this size constraint, collagen fibrils have to be processed beforehand and then cleaved into smaller units. Composed of insoluble fibres, collagens are resistant to most proteolysis mechanisms. Only an insignificant number of MMPs (MMP1, 2, 8, 13, 16) and cathepsins (mainly cathepsin K) display collagenolytic activities. MT1-MMP (MMP14) also exerts a collagenolytic activity with a key role in the regulation of collagen homeostasis in mice [41, 42]. These enzymatic activities lead to increased protein solubility that promotes the accessibility of cleavage sites to other collagenases. Thereby reduced in moieties, collagen fragments bound by the uPARAP/Endo180 LBR domain can be uptaken by the cell, routed first to the early endosomal compartment where they are dissociated from uPARAP/Endo180, and then to the late endosome/lysosomal compartment for intracellular degradation [24, 37, 38]. An alternative endocytic pathway of collagens relies on phagocytosis, which is uPARAP/Endo180 independent (Fig. 3) [43]. The key roles of uPARAP/Endo180 in collagen remodeling under physiological and pathological conditions are examined as follows.

Intriguingly, it has recently been demonstrated that uPARAP/Endo180 participates in the uptake and intracellular degradation of collectins, C-type lectins that contain triple-helical collagen-like domains. Collectins belong to the group of defense collagens such as mannose-binding lectin (MBL) and surfactant protein D (SP-D). Their expression is enhanced during infection, inflammation and fibrosis. Following tissue injury, collectins are temporarily deposited in extravascular sites where they manage (either promoting or limiting) inflammation and immune response [44]. The capability of uPARAP/Endo180 to uptake collectins is remarkably not shared by the MMR, the alternative endocytic receptor of collagen. The collectin uptake depends on specific residues within FNII domain that are not involved in collagen uptake and are absent in MMR. It can also rely on interaction with the CTLD2 for SP-D but not for MBL. This interesting study thus assigned a novel role for uPARAP/Endo180 in immunity. The demonstration of uPARAP/endo180-mediated clearance of collectins by fibroblasts in injured tissue, has extended the number of previously observed biological functions of this endocytic receptor to immune-regulatory roles [45, 46].

## uPARAP/Endo180: a member of a trimolecular complex with the urokinase-plasminogen activator (uPA) and its receptor (uPAR)

uPARAP/Endo180 acts, on certain cell types, as a co-receptor for the glycosyl phosphatidylinositol (GPI) anchored uPA-uPAR complex [47]. The uPA plays an indirect role in ECM degradation by contributing to the plasminogen activation cascade, converting plasminogen into plasmin that in turn activates pro-uPA, leading to a positive feed-back loop [48, 49]. Plasmin can, independently, breakdown numerous ECM components and further enhance degradation by activating several MMPs [50, 51]. This cascade of activation is negatively controlled by plasminogen activator inhibitors (PAI-1 and PAI-2), which inhibit uPA proteolytic activity and promote uPAR-uPA-PAI complex internalization [51–53]. In addition, uPARAP/Endo180 is requisite for the activation of signaling pathways when migrating cells sense the uPA gradient. Its expression promotes uPA-mediated filopodia formation and the chemotactic response towards an uPA gradient. The uPA-uPAR signaling pathway regulates the activation of two small Ras GTPases, Rac1 and Cdc42. Although the activation of this depends on uPARAP/Endo180 being expressed, it is independent of its internalization. Nonetheless, random cell motility evoked by uPARAP/Endo180 relies on receptor constitutive internalization, while the chemotaxis does not [47]. This dissimilarity may be explained by the fact that uPAR constitutive endocytosis is a clathrin-independent mechanism, unlike uPARAP/Endo180 which is not [50]. The uPARAP/Endo180, therefore, appears to be a versatile cell migration protein involved in ECM remodeling and in the regulation of uPAR/uPARAP-mediated cell chemotaxis (Fig. 3).

## uPARAP/Endo180, a regulator of a mechanotransduction pathway in cell contractility

The significance of uPARAP/Endo180 for mechanotransduction pathways that promote cell contraction has been well documented and it relies on the endocytic localization of uPARAP. uPARAP/Endo180-containing endosomes regulate contractile signals though the small GTPase RhoA, which, in turn, activates Rho-Rho kinase (ROCK) that phosphorylates a myosin light chain 2 (MLC2). Activation of the Rho–ROCK–MLC2 pathway leads to cell tail retraction, cellular junction remodeling and accordingly promotes cell migration. Notably, this effect has been reported to be independent of uPA-uPAR-uPARAP/Endo180 trimolecular complex formation [54] (Fig. 3).

## uPARAP/Endo180, a VEGFR-2/VEGFR-3 pathway regulator in lymphatic endothelial cell migration

The expression of uPAPAR/Endo180 by blood endothelial cells has already been identified in previous studies, although its role in vascular biology has only recently been documented. The results from this study unexpectedly revealed that uPARAP/Endo180 interferes with two vascular endothelial growth factor receptors (VEGFR-2 and VEGFR-3) in lymphatic endothelial cells (LEC) [8]. These data shed new light on a crucial role for this endocytic receptor in lymphangiogenesis in the formation of new lymphatic vessels associated with cancer progression and metastatic spread to lymph nodes and distant organs [55–59]. Vascular endothelial growth factor type C (VEGF-C) is the main lymphangiogenic factor that acts through its receptor (VEGFR-3) and leads to cell proliferation and migration towards the VEGF-C secretion source [47, 60, 61]. Localized at the cell leading edge toward VEGF-C gradient, uPARAP/Endo180 is essential for chemotactic response and cell guidance, as already described in non-lymphatic cell lines [47, 54, 62]. Silencing uPARAP/Endo180 in LEC was seen to drastically impair VEGF-C mediated cellular migration revealing that uPARAP/Endo180 may not regulate the direction of cell migration by itself, but, in fact, act as the first cog in the gearing represented here as an intracellular signaling network. Interestingly, in VEGF-C-stimulated LECs, uPARAP/Endo180 prevented the heterodimerization of VEGFR-2 and VEGFR-3. By acting as a gatekeeper between these two tyrosine kinase receptors, uPARAP/Endo180 contributes to modulating the downstream VEGFR-2 and VEGFR-3 signaling pathways. The reduced VEGFR-2/VEGFR-3 heterodimerization induced by uPARAP/Endo180 decreased VEGFR-2 signaling which, in turn, promoted the VEGFR-3-induced c-Jun N-terminal Kinase (JNK) Crk-II, and paxillin signaling pathway. The intertwining of these three proteins maintained the activate state of the Rho GTPase Rac, regulating cell migration (Fig. 3). Following deciphering of the uPARAP/Endo180 driven-cellular signaling pathway, related-phenotypes in experimental in vivo models were observed. In uPARAP-/- mouse, the lymphangiogenic response was enhanced when compared to the wild-type littermates, leading to an hyperbranched and dilated lymphatic vasculature with increased drainage capability [8].

## uPARAP/Endo180 in health and aging

Controlled ECM degradation is of central importance during embryonic development, growth and tissue repair. Collagen degradation is governed by an extracellular MMPs-mediated proteolytic pathway and an intracellular lysosomal degradation depending on uPARAP/Endo180-mediated endocytosis. Together with MMPs, uPARAP/Endo180 plays a key role in collagen clearance in normal physiology (tissue homeostasis and repair) [7, 63]. In healthy organisms, uPARAP/Endo180 is expressed by a restricted number of cell types, being mainly produced by mesenchymal cell types (fibroblasts, chondrocytes, osteoblasts and osteocytes), some macrophages and endothelial cells [12, 32, 64]. This receptor is largely limited to tissues characterized by an active matrix turnover such as the skin during wound healing [4] and in bone (Fig. 2), where uPARAP/Endo180 plays an important role in embryonic development and homeostasis. Mutations in the *MRC2* gene (c.2904_2905delAG and c.1906 T > C) are responsible for the Crooked Tail Syndrome observed in Belgian Blue cattle, which are characterized by increased muscle mass, a thickset head, scoliosis and short, but straight, fore limbs, [65, 66]. In mice, uPARAP/Endo180 genetic ablation leads to a significant decrease in the length of long bones associated with a trabecular bones reduction. Such impact is even more pronounced when associated with either a MMP-2 [67] or MT-MMP-1 [64] deficiency. In addition, implicated in this is uPARAP/Endo180, together with MMP-13 in the reversal phase of bone remodeling in humans [68] (Fig. 2). In mouse lungs, uPARAP/Endo180 deficiency leads to a higher pulmonary elastance related to impaired collagen internalization by pulmonary fibroblasts [69]. The collagen turnover decreases during the life span of mice and is associated with reduced cell-mediated collagen uptake and degradation by uPARAP/Endo180 with a reduction in expression from the MRC2 gene [70]. This impaired MRC2 expression contributes to age-related fibrosis (Fig. 2).

## uPARAP/Endo180 in disease

Fibroblasts are the main producers of collagens, and their endocytic receptors are central to tissue fibrosis and tumor fibrosis, the production of which is referred to as the desmoplastic reaction. An upregulation of uPARAP/Endo180 production has been reported in activated fibroblasts adjacent to collagen deposition in the liver [71], kidneys [72] and pulmonary fibrosis [69]. In carcinoma, the most common type of cancer, uPARAP/Endo180 is not expressed by epithelial tumor cells, but, rather instead, by cancer-associated fibroblasts (CAFs) within the tumor microenvironment [6]. The CAF-related uPARAP/endo180 implications in collagen remodeling and cancer progression has been well documented in the MMTV-PyMT breast cancer model [73]. uPARAP/Endo180 expressed by stromal cells associated with the cross-linking of collagen fibers by stromal-derived lysyl oxidase (LOX) regulates the migration of metastatic prostate cancer cells [74]. Interestingly, single-cell sequencing data revealed that the collagen endocytic receptor is produced, for the most part, by a specific CAF subset characterized by the expression of matrix components and matrix-modifying enzymes [75]. Findings from this study demonstrated the genetic ablation of uPARAP/Endo180 affects CAF contractility and viability, thereby limiting tumor growth and metastasis (Fig. 2). These data further support the pro-tumorigenic effects of matrix remodeling a CAF subset [76]. This differentiates it from other cancers where uPARAP/Endo180 can be produced by the cancer cells themselves and is the case in sarcomas such as osteosarcoma [35], some glioblastoma subsets [77] and triple-negative basal-like breast cancers [78]. Interestingly, in these types of cancer, epithelial tumor cells subjected by the epithelial-to-mesenchymal transition (EMT) often express higher levels of uPARAP/Endo180 [78]. The uPARAP/Endo180 expression could be dysregulated through the TGF-β pathway [77–80]. Importantly, and in contrast to its pro-tumor effect, uPARAP/Endo180 was seen to act as an EMT suppressor, when it binds the high glycosylated CD147 by its CTLD4 (Fig. 1). Indeed, either a downregulation of uPARAP/Endo180 or CD147, an anti-uPARAP/Endo180 antibody targeting CTLD4, or a dominant-negative GST-CTLD4 chimeric protein, promoted epithelial cell scattering, decreased E-cadherin and disrupted the adherens junctions [35].

## Conclusions and perspectives

Collectively, the data highlighted the pivotal role played by uPARAP/Endo180 in the extracellular matrix turnover, in fibrotic conditions and in the spread of malignant cancers [24, 73]. The significance of the soluble ectodomain of uPARAP/Endo180 as a biomarker for metastatic breast cancer has also been highlighted. The concentration of soluble uPARAP/Endo180 in plasma was found to be higher in patients displaying metastatic breast cancer than in patients with localized breast cancer, but lower in patients treated with bisphosphonates. This finding suggests that it could be used to monitor the effectiveness of this treatment [81]. A clinical study is ongoing to assess the efficacy of soluble uPARAP/Endo180 in a number of body fluids and urinary tissue factors as biomarkers of early malignancy in pancreatic cystic lesions (NCT036793). Further investigations still need to be carried out to assess the clinical interest of uPARAP/Endo180 as a biomarker for various cancer types.

In a therapeutic context, a monoclonal neutralizing antibody targeting uPARAP/Endo180 (epitope located in FNII) showed anti-tumor efficacy against experimental osteosarcoma (NCTC-2472 sarcoma) in mice. The strong protective effect observed in this model against bone destruction looks promising for establishing new treatment for this disease [82]. Another anti-uPARAP/Endo180 antibody (epitope located within the first three N-terminal domains of uPARAP, more particularly in either the CysR domain or CTLD1) has been conjugated to a drug (monomethyl auristatin E) [83]. Interestingly, a specific cytotoxicity in uPARAP-positive cancer cell lines of glioblastoma, sarcoma and leukemic origin was observed in vitro. The efficacy of this treatment was confirmed in vivo using a xenograft mouse model with human uPARAP-positive leukemic cells. Complete recovery of all mice, with no recurrent tumor growth or any observable adverse event was observed following intravenous administration of the antibody–drug conjugate [83]. The key implication of uPARAP in bone remodeling also suggested that it could be a target in metastatic bone disease caused by the metastatic spread of a primary tumor (such as, for instance, breast, prostate, lung and renal cancers). A mathematical model for the dysregulation of the uPARAP/Endo180 network (through a TGFβ signaling pathway in tumor and osteoblastic cells) has been developed to demonstrate its implication in bone destruction. This model could assist in future drug development in the context of metastatic bone disease [84]. The recently identified uPARAP/Endo180-mediated fibroblast function in the turnover of collectins supports the innovative concept that fibroblasts can regulate the innate immune system by evacuating collectins from injured tissue [45]. This novel immunological function of uPARAP/Endo180 offers new opportunities to investigate uPARAP/Endo180 contribution in various pathological conditions involving collectins (Fig. 2).

Beyond its role in collagen/collectin clearance, uPARAP/Endo180 increasingly appears as a membrane-associated molecule that interacts with multiple molecular partners. The current list of its interactors extends from the initial uPA-uPAR complex to EMMPRIN, and VEGF receptors [8]. Its unexpected implication in vascular biology is now emerging with a key role in lymphatic endothelial cell sprouting during pathological lymphangiogenesis. This recent discovery paves the way for future research in lymphatic biology and lymph node metastases. This protein could in particular offer new perspectives in the detection of and treatment of cancers and fibrotic disease. Further studies have yet to be carried out to take advantage of its potential in healthcare and, better understand and decipher its mechanism of action.

## Data Availability

Not applicable.
